# Exploring the complex relationship between women’s sanitation practices and household diarrhea in the slums of Nairobi: a cross-sectional study

**DOI:** 10.1186/s12879-019-3875-9

**Published:** 2019-03-11

**Authors:** Samantha Winter, Millicent Ningoma Dzombo, Francis Barchi

**Affiliations:** 1 Edward J. Bloustein School of Planning & Public Policy Rutgers, The State University of New Jersey, 33 Livingston Avenue, New Brunswick, NJ 08901 USA; 2 Department of Sociology and Social Work, University of Nairobi, Nairobi, Kenya

**Keywords:** Water, Sanitation and hygiene, Women, Informal settlements, Slums, Diarrhea, Developing countries, Kenya

## Abstract

**Background:**

Diarrheal disease kills over half a million people each year in sub-Saharan Africa; the majority are children under 5 years. About 58% of diarrhea cases are associated with poor water, sanitation, and hygiene—a critical issue for people living in informal settlements. In Kenya, 60% of Nairobi’s population lives in informal settlements; yet, there is a paucity of research exploring the relationship between water, sanitation and hygiene (WASH) conditions in these settlements and associated health outcomes.

**Methods:**

The study examines characteristics of women’s WASH behaviors and environments as potential factors associated with household diarrhea in Mathare Valley Informal Settlement in Nairobi using cross-sectional survey data collected from 550 women.

**Results:**

Approximately 17% of participants reported that at least one member of the household suffered from diarrhea in the previous 2 weeks—48% of the cases were children under five. Results from a logistic regression exploring factors associated with reports of household diarrhea suggest that women’s sanitation management strategies are associated with recent household diarrhea. Women who use toilets for defecation during the day, but rely on bags, buckets, or open defecation (OD) for urination during the day and for urination and defecation at night have over five time the odds of recent household diarrhea than women who use a toilet for all their sanitation needs. The odds of diarrhea were also higher for participants who walk up to 2 min to reach their toilets/sites for defecation and those who rely on water from taps inside buildings and plots. Odds were 62% lower for participants with clean toilets.

**Conclusions:**

Findings suggest that health targets to reduce the prevalence of diarrheal diseases in informal settlements may not be met unless particular attention is paid to the needs of women living in these environments.

## Background

Globally, diarrheal disease is the 9th largest cause of death among all ages and the 4th leading cause of mortality among children under the age of 5 years [1]. Over 40% of all-age deaths and more than 60% of under-5 mortality occurs in sub-Saharan Africa [1]. In 2015, in Kenya, childhood diarrheal disease accounted for 122 deaths per 100,000 children and over 790,000 disability-adjusted life years (DALYs)—almost one half of DALYs attributed to diarrheal diseases for Kenyans of all ages [1].

According to some estimates, poor water, sanitation, and hygiene (WASH) are responsible for approximately 58% of diarrheal-related deaths per year [2]. Other studies suggest the burden of disease associated with poor WASH may be even higher than those previously estimated [3]. Poor water, sanitation, and hygiene are particularly critical health concerns for people living in informal settlements where high population densities combined with a lack of formal waste management strategies, inadequate or absent sewers and storm drainages, and inconsistent and often contaminated water supplies increase residents’ risk of direct exposure to human feces and consumption of unsafe water [4]. Studies exploring the prevalence of diarrheal diseases in Kenya, especially, have found higher rates for children in urban areas, particularly informal settlements, than in other settings [5]. For example, statistics suggest that over 30% of children in informal settlements in Nairobi had experienced diarrheal episodes within the prior 2 weeks compared to about 13% in other parts of Nairobi and 17% in Kenya as a whole [6, 7]. In Kenya, 60% of Nairobi’s population lives in informal settlements [8]; yet, there is a paucity of research exploring the relationship between characteristics of residents’ sanitation and hygiene environments in these settlements and their health outcomes.

The few studies exploring the environmental and socioeconomic factors associated with prevalence of diarrhea in informal settlements in low- and middle-income countries have found important links between several WASH-related factors and the prevalence of diarrheal diseases in these settings. Several studies, for example, have found links between diarrheal infection in children and household drinking water sources [9, 10]; access to and conditions of sanitation [11, 12]; and caregiver knowledge and practices associated with hygiene and sanitation for diarrhea prevention [7, 9, 13].

Despite the growing body of literature suggesting important links between WASH-related factors and prevalence of diarrhea around the world, there is still a paucity of empirical research focused on these issues in informal settlements in East Africa, particularly Kenya. In addition, most research focuses exclusively on diarrhea cases in young children. While children under 5 years are the most affected by diarrheal-related morbidity and stunting, diarrhea is a health concern for residents of all ages, especially women [1]. Findings from a recent study in rural Kenya, for example, suggest that female adults have higher incidence of hospitalizations due to diarrheal infections than males [14].

Numerous studies in developing countries have suggested that women, as the managers of household WASH and the primary caretakers of children and elderly and disabled family members, play a critical role in ‘breaking the chain of contamination’ within their households [15–17]. As the primary caretakers of the home, women are responsible for supplying water for drinking, cooking, bathing, hygiene, and other domestic tasks [16]. Women are also primarily responsible for raising and educating children with regards to WASH and, frequently, for taking care of sick members of the household [16]. These roles and responsibilities put women in a critical position for establishing and maintaining safe and hygienic spaces within the household, ensuring that children form hygienic health- and sanitation-related habits, and helping to minimize illness from preventable diseases [16]. For example, findings from several studies suggest that women’s sanitation behaviors and their ability to access sanitation is linked to their children’s sanitation behaviors, especially for young children [18, 19]. Findings from a 2016 qualitative study suggest that one of the factors that influences women’s sanitation behaviors in informal settlements in Nairobi is the need to set an example and/or be a role model for their children’s sanitation practices [19]. Other studies report that women with small children often take their children with them when they leave the home to access sanitation and/or collect water [20–22]—suggesting that the mother’s WASH-related behaviors may be linked to their children’s exposure to different environments and, consequently, their health [16, 18]. Additionally, if a woman lacks the ability to pay to use a public toilet for herself, she is also unlikely to be able to pay for her children to use the public facility—suggesting that both she and her children have to find alternative methods of disposal, such as the use of bags/buckets in the home or open defecation (OD), to meet their sanitation needs [18, 23]. Lastly, women are typically responsible for two other potential sources of household diarrhea: cooking and handling of food and collecting and managing household water supplies [24]. Despite suggestions of a link between women’s WASH-related environments or behaviors and household health, there are few studies focusing on this phenomenon. This study is a step towards filling a piece of this gap by examining associations between characteristics of women’s sanitation and hygiene knowledge, behaviors and environments and occurrences of household diarrheal disease in an informal settlement in Nairobi, Kenya.

## Method

### Data and sample

Cross-sectional data for this study were collected in 2016 in Mathare Valley Informal Settlement in Nairobi Kenya. Mathare is one of the oldest and largest informal settlements in Nairobi. It is home to approximately 250,000 residents living in an area about 3-km^2^ [25]. The study from which the data were taken focused on women’s sociodemographic information, access to and utilization of water and sanitation, health, and neighborhood dynamics in Mathare. Information was gathered from 550 women (50 from each of Mathare’s 11 main villages). Households were randomly selected using a grid and random point sampling in ArcMap [26], and individual women were selected from each household using Kish methodology [27]. In order to participate in the study women were required to be over 18 years, provide consent to participate, and speak either English or Swahili. The surveys lasted about 60 min.

### Measures

Variables for this study were created from responses to structured questions on the 550 household surveys. The dichotomous outcome variable (1 = “yes”; 0 = “no”) was created from women’s answers to the question, “in the last 2 weeks, has anyone in your household had diarrhea?” where diarrhea was defined as passing stool 3 times or more in 24 h whether watery, bloody, mucoid or water-wash like.

A number of potential diarrhea-related WASH factors were included in the models. Women in this study were asked questions about their primary toilet/site for urination and defecation during the day and during the night. Responses to these questions were used to identify and characterize five common sanitation utilization profiles for women, which have been previously described in a separate paper [17]. The common sanitation profiles included:

**Table:** 

Sanitation Profiles	
1. Day: toilet for urine/feces; Night: toilet for urine/feces	
2. Day: toilet for urine/feces; Night: bags/buckets/OD for urine/feces	
3. Day: toilet for feces, bags/buckets/OD for urine; Night: bags/buckets/OD for urine/feces	
4. Day: toilet for urine/feces; Night: toilet for feces, bags/buckets/OD for urine	
5. Day: bags/buckets/OD for urine/feces; Night: bags/buckets/OD for urine/feces	

Primary drinking-water source is often connected to diarrheal disease [1]; thus, responses to a question about women’s primary source for drinking water were collapsed into a four-category water source variable (1. tap inside home, building, or plot; 2. shared tap outside building or plot; 3. public tap or well; or 4. tanker or vendor). Questions to measure WASH knowledge, attitudes, and practices (WASH-KAP)—factors, e.g. knowledge of the primary vectors of infection, hand-washing behaviors, and sanitation-related hygiene, associated with diarrhea disease [2, 7, 13, 28]—were adapted from questionnaires developed and used in India and South Africa [29, 30]. These included: 1) a measure to indicate a woman’s knowledge about WASH behaviors and health; 2) a variable to assess whether or not a woman regularly treated her drinking water; 3) a measure to represent whether a woman regularly used soap/a disinfectant/sanitizer to wash her hands; and 4) a variable to measure whether a woman used tissue for anal cleansing (the most common practice) compared to other methods of cleansing.

The following diarrhea-associated factors related to a woman’s primary toilet/methods of urine/feces disposal were also included: 1) whether the respondent felt her toilet/site for urination/defecation was clean [7, 31]; 2) whether the respondent’s toilet/site for urination/defecation regularly had running water [18]; 3) whether a respondent’s toilet/site for urination/defecation had a location for washing hands [18]; 4) the walk time to reach the toilet/site for disposal (0 min, less than 1 min, 1–2 min, 3–4 min, and 5 or more minutes) [11]; and 5) the approximate number of people using the toilet [31]. Finally, several core socio-demographic variables including whether the respondent had children, household income, whether the respondent was married, and respondents’ age and education were also included.

### Analysis strategy

Bivariate analyses were run to examine the relationship between recent diarrhea in the household and WASH-related and sociodemographic variables. Variables were tested for multicollinearity using variance inflation factors (VIFs); values were all less than or equal to 3.10. Bivariate analyses suggested that all WASH-related factors were significantly associated with recent household diarrhea except primary water source, use of disinfectant (e.g. soap, sanitizer or ash), and WASH knowledge score. While these three variables were not significantly associated with recent household diarrhea at the bivariate-level, they were included in subsequent regressions as important covariates or potential WASH factors commonly associated with diarrheal disease in the literature [2, 32]. Women’s reports of diarrhea in the household in the previous 2 weeks was regressed on all potential diarrhea-related WASH factors using binary logistic regression in Stata Statistical Software, v.15 [33]. Standard errors were clustered by village to account for the fact that 50 surveys were collected in each of Mathare’s 11 villages. Although missing values were minimal on individual variables (less than 3%), the missing data were imputed using a single imputation method [34].

## Results

### Sociodemographic statistics

Descriptive statistics for the sample are presented in Table 1. Findings suggest that 16.5% of the women in the sample reported diarrhea in the household in the 2 weeks leading up to the survey, and of those cases, 48% were children under the age of 5 years. Over half of the sample was married and over 81% of the women had at least one child. About three-quarters of the women had monthly household incomes of less than KES 10,000 per month (USD 100). Over half the women reported good health. During the day, about half the women reported using a public toilet, 20% reported using a plot or building toilet, and about 30% reported using bags or buckets at home. At night, over two-thirds of the women reported using bags or buckets at home and about 19% reported using plot or building toilets. The majority of women (57%) use a public tap or well for their primary drinking water source followed by an outside tap (16%), tankers or vendors (14%), or a tap inside their home/building (14%). Out of a possible score of 20, the average score on the WASH -KAP scale was 13 (SD = 4.1).

**Table 1:** Demographic statistics (*n = 550*)

	Total		Recent Diarrhea		
	Freq	%	No	Yes	Χ^2^
Outcome					
Recent diarrhea in household	91	16.5	459	91	–
Proportion children < 5 years	44	8.0	506	44	–
Socio-Economic Factors					
Age (*continuous*)	32.2 (8.36)	–	–	–	1.03**†
Education					1.79
Less than complete primary	102	18.5	86	16	
Completed primary	136	24.7	112	24	
Some secondary	122	22.2	98	24	
Completed secondary	190	34.5	163	27	
Employed	250	45.5	198	52	6.01*
Married	299	54.4	253	46	0.64
Has children	449	81.6	363	86	12.05**
Household income					12.27**
Less than Ksh 5000/month (US$50)	138	25.1	118	20	
Between Ksh 5000-10,000/month	278	50.5	218	60	
More than Ksh 10,000/month	134	24.4	123	11	
Toilet descriptors					
Sanitation Profiles					46.67***
Profile 1: Day - toilet for urine/feces; night - toilet for urine/feces	137	24.9	122	15	
Profile 2: Day - toilet for urine/feces; night - bags/buckets/OD for urine/feces	148	26.9	129	19	
Profile 3: Day - toilet for feces, bags/buckets/OD for urine; night - bags/buckets/OD for urine/feces	120	21.8	76	44	
Profile 4: Day - toilet for urine/feces; night - toilet for feces, bags/buckets/OD for urine	66	12.0	58	8	
Profile 5: Day - bags/buckets/OD for urine/feces; night - bags/buckets/OD for urine/feces	79	14.4	74	5	
Relies on public toilet at least once in a 24-h period	279	50.7	217	62	13.21***
Water source					
Primary water source					2.02
Tap inside home/building	74	13.5	62	12	
Tap outside home/building	89	16.2	70	19	
Public tap or well	312	56.7	265	47	
Tanker/vendor	75	13.6	62	13	
Toilet hygiene and accessibility					
Toilet(s) are clean	340	61.8	293	47	4.78*
Toilet(s) have running water/water for hygiene	184	33.5	163	21	5.27*
Maximum number of people sharing toilet(s) (*continuous*)	152.4 (219.37)	–	–	–	1.001**†
Maximum walk time to toilet(s)					12.64**
Does not go outside	146	26.5	132	14	
2 min or less	212	38.5	164	48	
Between 3 and 4 min	85	15.5	69	16	
At least 5 min	107	19.5	94	13	
WASH knowledge and practices					
Uses disinfectant (e.g. soap) to wash hands	453	82.4	378	75	0.00
Treats drinking water	179	32.5	161	18	8.09**
Uses something other than tissue for anal cleansing	236	42.9	183	53	10.46**
WASH knowledge score (*continuous*)	13.1 (4.08)	–	–	–	1.05†

†*p* < 0.1; *p < 0.05; **p < 0.01; ***p < 0.001

### Women’s knowledge about water, sanitation, and hygiene (WASH – KAP)

Women in this survey were asked several general open-ended questions (multiple responses were allowed) about WASH and health. A summary of the responses are reported in Table 2. About half of the women in this study (49%) identified diarrhea as the most common effect from drinking unsafe water followed by cholera (34%) and typhoid (31%). While over half of the sample (56%) felt that everyone is affected by diarrhea, just under 48% reported that young children are the most affected. In response to the question, ‘how does a person get diarrhea,’ over 80 % of women identified dirty hands (82%), germs (85%), dirty food (86%), poor hygiene (83%), and dirty water (82%) as the primary causes of diarrhea. Over 60 % also identified flies (69%) and OD (63%) as causes. Ninety percent of women reported drinking clean/safe water as the primary practice to prevent getting diarrhea. Washing food before it is eaten (87%), using a toilet (82%), treating water (79%), washing hands with water and soap/ash (78%), covering food (78%) and avoiding OD and flying toilets (70%) were also common practices identified to prevent diarrhea. In response to the following question, “kindly give me the key times you should wash your hands,” almost 99% of the women said before eating and close to 92% said after eating. After toilet use (88%), after defecation (76%), after handling babies feces/changing a diaper (73%), and after handling rubbish (61%) were also key times.

**Table 2:** Women’s Water, Sanitation, and Hygiene (WASH) Knowledge (*n = 550*)

Question	Summary of Responses	Total Population		Proportion with Recent Diarrhea		
		Count	%	Count	% of total sample	% for each category
What are the most common effects of drinking unsafe water (more than one answer is acceptable)?	Vomiting	39	7.1	1	0.2	2.6
	Stomach ache	42	7.6	6	1.1	14.3
	Amoeba	64	11.6	13	2.4	20.3
	Infection	10	1.8	1	0.2	10.0
	Typhoid	168	30.5	36	6.5	21.4
	Cholera	185	33.6	42	7.6	22.7
	Diarrhea	271	49.3	31	5.6	11.4
Who do you think is most affected by diarrhea (more than one answer is acceptable)?	Adults	1	0.2	0	0.0	0.0
	Children (under 5)	263	47.8	38	6.9	14.4
	Elderly people	25	4.5	6	1.1	24.0
	Pregnant women	1	0.2	0	0.0	0.0
	Adult men	0	0.0	0	0.0	0.0
	Adult women	2	0.4	0	0.0	0.0
	Everyone	309	56.2	55	10.0	17.8
	Other (specify)	1	0.2	1	0.2	100.0
How does a person get diarrhea (more than one answer is acceptable)?	Dirty hands	450	81.8	80	14.5	17.8
	It’s part of a child’s growth	60	10.9	8	1.5	13.3
	Black magic/witchcraft	32	5.8	5	0.9	15.6
	Germs	467	84.9	81	14.7	17.3
	Dirty food	473	86.0	81	14.7	17.1
	Poor hygiene	454	82.5	79	14.4	17.4
	Flies	379	68.9	64	11.6	16.9
	Dirty water	449	81.6	80	14.5	17.8
	Open defecation/bags/buckets	347	63.1	64	11.6	18.4
	Other (specify)	11	2.0	0	0.0	0.0
How can you prevent yourself and your family members from getting diarrhea (more than one answer is acceptable)?	Do not know	6	1.1	2	0.4	33.3
	Drink clean water	492	89.5	83	15.1	16.9
	Wash your food before eating	476	86.5	85	15.5	17.9
	Cook foods	344	62.5	46	8.4	13.4
	Toilet use	450	81.8	82	14.9	18.2
	Treating water	434	78.9	78	14.2	18.0
	Washing hands with water and soap/ash	428	77.8	77	14.0	18.0
	Covering food	428	77.8	71	12.9	16.6
	Avoiding open defecation/bags/buckets	386	70.2	68	12.4	17.6
	Going to a traditional healer	18	3.3	3	0.5	16.7
	Prayer	71	12.9	9	1.6	12.7
	Storing water safely	252	45.8	54	9.8	21.4
	Other (specify)	5	0.9	0	0.0	0.0
Kindly give me the key times you usually wash your hands (more than one answer is acceptable)?	Never	0	0.0	0	0.0	0.0
	Before eating	543	98.7	89	16.2	16.4
	After eating	503	91.5	85	15.5	16.9
	After defecation	417	75.8	76	13.8	18.2
	After urination	300	54.5	59	10.7	19.7
	After toilet use	485	88.2	81	14.7	16.7
	After handling animals	148	26.9	12	2.2	8.1
	After handling babies’ feces/diaper	402	73.1	80	14.5	19.9
	After handling rubbish	334	60.7	66	12.0	19.8
	When they are visibly dirty	323	58.7	53	9.6	16.4
	After handling manure	126	22.9	11	2.0	8.7
	Other (specify)	5	0.9	1	0.2	20.0

### Diarrhea-related water, sanitation, and hygiene factors

Results from the bivariate analyses of WASH-factors and recent household diarrhea are presented in Table 1 and findings from the logistic regression are summarized in Table 3. Results suggest that women with children had over four times the odds of reporting recent diarrhea in the family (OR = 4.3, 95% CI = 1.42–12.75, *p* = .015) than women without children. Additionally, women in sanitation profile 2—those who rely on a toilet for defecation during the day, but utilize bags, buckets, or OD for urination during the day and for both urination and defecation at night—had over five times the odds of reporting at least one case of diarrhea in the family in the previous 2 weeks than women in other sanitation profiles (OR = 5.2, 95% CI = 2.21–12.21, *p* = 0.002).

**Table 3:** Factors associated with reports of diarrhea in the household in the last 2 weeks (*n = 550*)

	Odds Ratio	*p*-value	CI 95%
Socio-Economic Factors			
Age (*continuous*)	1.03	0.167	0.985–1.077
Education (less than complete primary)			
Completed primary	0.86	0.771	0.274–2.691
Some secondary	1.18	0.737	0.402–3.478
Completed secondary	1.11	0.786	0.475–2.603
Employed	2.12	0.069	0.931–4.809
Married	0.82	0.469	0.447–1.489
Has children	4.26	0.015	1.424–12.753
Household income (ref: less than 5000/month)			
Between Ksh 5000-10,000/month (US$50–$100)	1.96	0.156	0.739–5.184
More than Ksh 10,000/month (>US$100)	0.80	0.714	0.207–3.06
Toilet descriptors (ref: Profile 1: Day - toilet for urine/feces; nights - toilet for urine/feces)			
Profile 2: Day - toilet for urine/feces; night - bags/buckets/OD for urine/feces	1.57	0.212	0.737–3.354
Profile 3: Day - toilet for feces, bags/buckets/OD for urine; night - bags/buckets/OD for urine/feces	5.20	0.002	2.212–12.211
Profile 4: Day - toilet for urine/feces; night - toilet for feces, bags/buckets/OD for urine	0.74	0.322	0.395–1.4
Profile 5: Day - bags/buckets/OD for urine/feces; night: bags/buckets/OD for urine/feces	0.23	0.128	0.032–1.657
Water source			
Primary water source (ref: public tap/well)			
Tap inside home/building	3.79	0.018	1.329–10.818
Tap outside home/building	3.02	0.057	0.959–9.48
Tanker/vendor	1.72	0.295	0.578–5.095
Toilet hygiene and accessibility			
Toilets are clean	0.38	0.007	0.198–0.723
Toilets have running water/water for hygiene	0.48	0.116	0.187–1.24
Maximum number of people sharing toilet(s) (*continuous*)	1.00	0.147	0.998–1
Maximum walk time to toilet(s) (ref: does not go out)			
2 min or less	2.04	0.009	1.249–3.336
Between 3 and 4 min	1.41	0.533	0.427–4.68
At least 5 min	0.58	0.246	0.218–1.55
WASH knowledge and practices			
Uses disinfectant (e.g. soap) to wash hands	0.54	0.235	0.179–1.609
Treats drinking water	0.56	0.064	0.306–1.042
Uses something other than tissue for anal cleansing	1.15	0.682	0.54–2.472
WASH knowledge score (*continuous*)	1.08	0.130	0.972–1.21

Women who relied on water from taps inside their homes had higher odds of reporting at least one case of recent diarrhea in the family compared to women who used public taps or wells (OR = 3.8, 95% CI = 1.33–10.82, *p* = 0.018). Women who reported that their toilets were clean had 62% lower odds of reporting a recent case of diarrhea in the family (95% CI = 0.20–0.72, *p* = .007) compared to women who reported their toilets were dirty/neither clean nor dirty. Women who had to go outside their homes to access a place to urinate/defecate, but walked 2 min or less had double the odds of reporting a case of recent diarrhea among members of the household compared to women who did not go outside their house to access a toilet/site for urination/defecation (OR = 2.0, 95% CI = 1.25–3.34, *p* = 0.009).

## Discussion

The purpose of this study was to examine associations between characteristics of women’s sanitation and hygiene knowledge, behaviors and environments and recent cases of diarrhea in the Mathare Valley informal settlement in Nairobi, Kenya. Diarrhea is one of the leading causes of death among children under 5 years and a serious contributor to the global burden of disease for people of all ages, especially in informal settlements in sub-Saharan Africa. Results of this study provide important information for developing better and more targeted intervention and policy strategies to prevent diarrhea in these rapidly expanding settlement environments.

Several findings from this study are consistent with evidence from other studies exploring factors associated with recent diarrheal infections in urban and peri-urban informal settlements. For example, women who reported that their toilets were clean in this study had lower odds of reporting recent diarrhea in the household. This is consistent with findings from other studies [11, 12] suggesting that the cleanliness of toilets is a key factor associated with diarrhea. Studies suggest this is particularly true in informal settlements where toilets are often shared between many families and the locus of responsibility for cleaning these toilets is not often clearly defined [35].

Primary water source—another common factor associated with diarrheal disease—was also associated with recent household diarrhea in this study. Women who reported using taps inside their buildings, plots, or houses had higher odds of recent household diarrhea than women who rely primarily on public wells or taps. Recent literature suggests that while public water kiosks and taps are usually operated by formally licensed providers in informal settlements in Nairobi and, consequently, are regulated, private vendors, e.g. households and/or landlords who supply water to their tenants in plots or buildings, are usually unregulated [36]. In light of literature focused on water providers in informal settlements in Kenya, the findings from this study may suggest that private water sources, e.g. those within households, plots, or buildings, may not be regulated and, consequently, unsafe for drinking.

Several previous studies have also found that the number of children in a household increases the risk of recent diarrhea [5, 10, 37, 38]. Findings from this study suggest that women *with* children had higher odds of reporting at least one case of diarrhea in the household in the preceding 2 weeks, but, when controlling for other factors, the *number* of children did not have a significant association (results not shown).

Women in sanitation profile 2—those who rely primarily on toilets for defecation during the day, but use bags, buckets, and OD for urination during the day and for urination and defecation at night—had significantly higher odds of reporting acute diarrhea by at least one member of their household in the preceding 2 weeks. While it is difficult to determine precisely why women in this sanitation profile had higher odds of recent diarrhea among members of their household, it could be related to the type of facility these women use. All but one of the women in this profile, for example, rely on public toilets for defecation during the day (99.2%). In order to test this hypothesis, we ran a separate logistic regression model (results presented in Appendix) to examine the relationship between women who relied on public toilets at least once in a 24-h period and recent diarrhea in the household. Findings from this analysis suggest that women who use public toilets have higher odds of reporting recent diarrhea in the household compared to women who do not use public facilities (OR = 2.3, 95% CI = 1.54–3.3, *p* = 0.001). The magnitude and significance of the other covariates and WASH-related factors in the model were similar to those in the primary logistic regression (See Appendix).

Interestingly, the number of people sharing women’s toilets/sites for urination/defecation was not significantly associated with recent diarrhea in the family in the regression analysis, which is inconsistent with evidence from previous studies [11, 38]. The association between use of public toilets, the majority of which are used by more than 100 people in Mathare (77%), and recent household diarrhea may indicate that the type and management of a toilet and the relationship between the people using it may be of more importance than just the number of people sharing it. Finally, results from this study also suggested that distance (walk time) to reach a toilet was associated with higher odds of recent diarrhea—a finding that is consistent with other studies [11]. Walk time or distance to a toilet may also be an indication of the type of toilet facility on which women are relying. For example, mid-range walk times likely indicate the use of plot/building toilets or nearby public toilets while longer walk times likely indicate use of further public toilets or OD.

Interestingly, women in this study who relied exclusively on OD and/or bags or buckets in their homes and disposing of feces in open drainages outside their homes, i.e. those in sanitation profile 5, did not have higher odds of reporting recent diarrhea compared to women using toilets as all times (sanitation profile 1) as has been found in previous studies [12]. This finding, however, may point to an important disconnect between individual- and public-health when it comes to sanitation. According to McGranahan [39] an individual’s sanitation strategies can, in principal, be more clean, hygienic, safe, and private than other alternatives from the user’s perspective, but still impose a heavy health burden onto others and the community as a whole. For example, women in Mathare may feel that OD and/or use of bags/buckets is a safer, more manageable, cleaner, or even hygienic option than a public or shared toilet; however, as soon as the raw sewage is left in the open or emptied into open drainages, everyone in the community is likely to be at greater risk. While exclusive use of OD or bags/buckets in the home may not increase the odds of individuals in that household getting diarrhea because, for example, women may work hard to ensure that the process is as hygienic as possible, women who rely exclusively on these strategies also do not show significantly lower odds of household diarrhea compared to women who utilize toilets at all times. This could be because the health burden of disposing of raw sewage in the environment is shared, more or less equally, by all in the local community.

According to literature, caregiver knowledge related to WASH and health can be an important protective factor to prevent diarrhea [7, 9]; yet, evidence of the association between WASH and health knowledge and diarrhea are sometimes mixed [40, 41]. Some studies suggest, for example, that high levels of overall WASH and health knowledge do not always lead to better diarrheal prevention behaviors [40, 41]—findings which are consistent with findings in this study. For example, over two-thirds of the women in this study identified OD and/or use of bags and buckets (emptied into open drainages) as a primary cause of diarrhea and 70% said avoiding OD and/or use of bags and buckets was a key diarrhea prevention strategy; yet, close to 69% of the women reported that they rely on bags or buckets for urination/defecation at night and an additional 6% reported defecating in the open at night. One explanation for this findings might be, as evidence from other studies corroborates [42, 43], that there is a knowledge-behavior gap when it comes to issues of WASH. Women may know, for example, that use of OD and/or bags and buckets is linked to poor health outcomes, but abandoning these practices may be hindered by additional more-pressing factors that prevent women, and their children and family members, from accessing clean water, safe sanitation, and or products for safe hygiene [17, 44]. For example, several recent studies have provided evidence that women in sanitation-poor environments, e.g., informal settlements, often face a number of gender-specific barriers to access sanitation such as lack of privacy and dignity [18, 45, 46] and sexual violence and harassment associated with having to rely on community toilets or sites for OD at night or during menstruation [22, 45–48]. In this study, for example, 80% of women reported that it is not safe to go alone to a toilet at night—a barrier that may need to be addressed if women and, more than likely, their children are to consider abandoning the use of OD or bags and buckets, especially at night. An alternative explanation might be that women’s WASH and health knowledge influences their decisions to use methods in their homes because they perceive them to be more hygienic than the alternatives. Literature suggests that shared sanitation in informal settlements may be associated with poor health outcomes, e.g. diarrhea [31, 35, 49–51]; women may be using bags, buckets, or OD in or near the home not out of ignorance of the health risks, but rather because they perceive such practices to be healthier options than using shared sanitation facilities regardless of any greater environmental or public health implications.

Overall, findings from this study suggest that a number of characteristics of women’s sanitation and hygiene knowledge, behaviors and environments are associated with recent cases of household diarrhea in Mathare. Findings suggest that women in this settlement are knowledgeable about WASH and health and adopt reasonable sanitation-management strategies in light of the numerous challenges they face in these settlements. Some of their sanitation-management strategies, e.g. relying on OD or using bags and buckets that are subsequently emptied into open drainages, may have serious public and environmental health implications; thus, it is critical to consider reasonable solutions to help women have more and better options for sanitation management. Given that findings from the research suggest women in Mathare are knowledgeable about WASH and health, solutions to sanitation issues in this settlement should focus on addressing other external factors that continue to limit women’s ability to access sanitation, e.g. safety and privacy. A growing number of sanitation interventions have been implemented in Mathare in recent years, such as Sanergy’s Fresh Life toilets, Grand Challenge Canada’s funded Banza toilets, and/or National Youth Service’s (NYS) slum improvement project toilets, to name a few, but little is known about the effect of these interventions on women’s ability to consistently access sanitation throughout a 24-h period, changes in women’s sanitation management strategies or on sanitation-related health outcomes like diarrhea. Findings from a recent article focused on women’s solutions to sanitation challenges in Mathare, suggest that strategies aimed at supporting women’s efforts for collective action around issues of sanitation and co-production efforts between landlords and governments may help women have more and better options to manage household sanitation more safely and, consequently, to reduce household diarrhea [23]. These collective action and co-production strategies are also in-line with the WHO’s recent Guidelines on Sanitation and Health, which encourage sanitation strategies and interventions that combine government leadership, oversight, monitoring, and potential funding with locally delivered services [52].

There were several limitations to this study. For example, we relied on a self-reported measure of diarrhea provided by only one female resident of each household. While self-reported measures of two-week diarrhea provided by the primary caregiver (usually the mother) are common in studies assessing the prevalence of diarrhea among children under the age of 5 years, this study neither focused exclusively on under-5 children nor did it require that the female study participant be the primary caregiver in the household. Thus, the women in this study may not have been able to provide an accurate account of diarrhea for all members of the household, nor are their reported sanitation practices necessarily the same as those of other members of the household. Second, while this study included many of the common socio-economic, environmental, and WASH factors assessed in similar diarrhea prevalence studies, the list is not comprehensive. We did not, for example, include factors such as water quality measures, observed hygiene measures (e.g., presence of flies in house/on food/in toilets, observed feces in toilet facilities or in open drainages, sewage conditions, or garbage disposal methods), or medical factors (e.g., fever, stunting, and dehydration)—variables that have shown to be important risk or protective factors in other studies [10–12].

## Conclusion

This study explored socio-economic, environmental, and WASH-related factors associated with recent diarrheal diseases in informal settlements in Nairobi, Kenya. While many of the findings are consistent with previous literature that emphasizes the importance of access to clean/safe sources of drinking water and nearby sanitary toilet facilities as protective factors for diarrhea, this study also suggests that there exists an important gap between WASH knowledge and behavior in this setting. Even though the majority of women in this study were able to correctly identify key times to wash their hands, common causes of diarrhea, and strategies to prevent diarrhea, they still engaged in behaviors that are associated with poor individual and/or public health outcomes. Findings from this study suggest that global health targets to reduce the prevalence of diarrheal diseases will not be met unless particular attention is paid to the needs of women living in informal settlements. For example, this study provides an evidence base for the profound role that women’s sense of safety, fear of violence, and concern over the cleanliness of facilities play in their sanitation behaviors. Such externally driven factors will not be addressed solely through WASH and health education programs aimed at individuals. Interventions must also address the underlying, external factors that drive the gap between knowledge and behavior in these communities.

## Appendix

**Table 4:** Public toilet associated with reports of diarrhea in the household in the last 2 weeks (*n = 550*)

	Odds Ratio	*p*-value	CI 95%
Socio-Economic Factors			
Age (*continuous*)	1.03	0.219	0.981–1.076
Education (less than complete primary)			
Completed primary	0.88	0.808	0.279–2.771
Some secondary	1.22	0.682	0.424–3.521
Completed secondary	1.01	0.989	0.4–2.526
Employed	2.17	0.046	1.016–4.613
Married	0.85	0.520	0.485–1.477
Has children	3.93	0.024	1.243–12.455
Household income (ref: less than 5000/month)			
Between Ksh 5000-10,000/month (US$50–$100)	2.15	0.052	0.993–4.636
More than Ksh 10,000/month (>US$100)	0.77	0.668	0.207–2.866
Toilet descriptors			
Public toilet	2.25	0.001	1.542–3.284
Water source			
Primary water source (ref: public tap/well)			
Tap inside home/building	3.78	0.023	1.254–11.384
Tap outside home/building	2.83	0.054	0.977–8.173
Tanker/vendor	1.67	0.315	0.566–4.958
Toilet hygiene and accessibility			
Toilet(s) are clean	0.44	0.014	0.241–0.814
Toilet(s) have running water/water for hygiene	0.53	0.209	0.186–1.517
Maximum number of people sharing toilet(s) (*continuous*)	1.00	0.353	0.999–1.002
Maximum walk time to toilet(s) (ref: does not go out)			
2 min or less	3.28	0.001	1.927–5.59
Between 3 and 4 min	2.15	0.157	0.705–6.586
At least 5 min	0.95	0.853	0.549–1.657
WASH knowledge and practices			
Uses disinfectant (e.g. soap) to wash hands	0.69	0.413	0.262–1.815
Treats drinking water	0.55	0.068	0.285–1.056
Uses tissue for anal cleansing	1.22	0.551	0.594–2.513
WASH knowledge score (*continuous*)	1.10	0.059	0.996–1.216

## Abbreviations

CI: Confidence interval
DALYs: Disability-adjusted life years
KES: Kenya shilling
NACOSTI: National Commission for Science, Technology and Innovation
NYS: National Youth Service
OD: Open defecation
OR: Odds ratio
UNDESA: United Nations Department of Economic and Social Affairs
USD: United States dollar
VIF: Variance inflation factor
WASH: Water, sanitation, and hygiene
WASH-KAP: Water, sanitation, and hygiene knowledge, attitudes, and practices
WHO: World Health Organization
